# Association between admission Braden Skin Score and delirium in surgical intensive care patients: an analysis of the MIMIC-IV database

**DOI:** 10.3389/fneur.2025.1555166

**Published:** 2025-04-14

**Authors:** Meiling Shang, Ailing Shang, Yu Xu

**Affiliations:** ^1^Department of Critical Care Medicine, West China Hospital, Sichuan University, Chengdu, China; ^2^West China School of Nursing, Sichuan University, Chengdu, China; ^3^Department of Emergency and Critical Care Medicine, Chengdu BOE Hospital, Chengdu, China

**Keywords:** Braden Skin Score, delirium, surgical intensive care unit, MIMIC-IV database, risk factors

## Abstract

**Background:**

The Braden Skin Score (BSS), a tool for assessing pressure ulcers, is increasingly recognized for its prognostic value in various disorders. However, its link to critical delirium in surgical patients remains understudied. This study aimed to explore the association between BSS upon admission and the risk of delirium in SICU patients.

**Methods:**

This retrospective observational cohort study used data from the Medical Information Mart for Intensive Care (MIMIC)-IV database. The primary outcome was incidence of delirium. Feature importance of BSS was initially assessed using a machine learning algorithm, while restricted cubic spline (RCS) models and multivariable logistic analysis evaluated the relationship between BSS and delirium. Additionally, Kaplan–Meier analysis and mediation analysis were conducted to explore interactions among BSS, delirium, and short-term mortality.

**Results:**

A total of 4,899 patients were included in the study, among whom 1,491 were diagnosed with delirium. The Boruta algorithm identified BSS as a significant predictor of delirium occurrence. RCS models demonstrated a non-linear positive relationship between BSS and delirium. Based on RCS curves, the optimal threshold for BSS was established at 16, thereby categorizing participants into two groups: those with BSS < 16 and those with BSS ≥ 16. Multivariable logistic regression analysis revealed that lower BSS was positively correlated with an increased risk of delirium. These findings exhibited robust consistency across subgroup analyses and sensitivity analyses. Furthermore, patients in lower BSS groups had a higher 90-day mortality, with delirium mediating an indirect effect on this outcome.

**Conclusion:**

The low BSS was independently associated with an increased risk of delirium in critically ill surgical patients. Patients exhibiting a BSS below 16 demonstrated heightened susceptibility to the onset of delirium, thereby necessitating vigilant monitoring and timely intervention. Larger prospective studies are needed to confirm these findings.

## 1 Introduction

Delirium is a condition that presents with acute dysfunction of the central nervous system, primarily characterized by disturbances in consciousness and cognitive function (1, 2). In the surgical Intensive Care Unit (SICU), it has been observed that the incidence of delirium can reach as high as 60% (3). Compared to non-surgical ICUs, surgical ICU patients face unique risk factors for delirium, including specific anesthetic agents, pain management techniques, and longer surgery durations (3, 4). Delirium is strongly linked to negative clinical outcomes such as extended mechanical ventilation, length of stay, excess death, and diminished long-term quality of life (4). Despite recent proposals for bundled management strategies aimed at preventing delirium, both the incidence and mortality rates associated with this condition have shown only limited decline over the past few years (5). This underscores the critical importance of identifying populations at high risk for delirium to facilitate the development of early preventive strategies.

The Braden Skin Score (BSS), collaboratively developed by Barbara Braden and Nancy Bergstrom in 1987, is designed to assess the risk of pressure ulcer in patients (6). It evaluates susceptibility based on six factors: sensory perception, moisture, activity level, mobility, nutritional status, and friction/shear. The total BSS varied between 6 and 23, with lower scores indicating an increased risk of pressure injuries. Numerous studies have demonstrated that the BSS exhibits strong sensitivity in both evaluating the progression and preventing pressure injuries, leading to its widespread use in clinical practice (7, 8). For critically ill patients, BSS is a bedside nursing assessment conducted early in hospitalization to predict the risk of pressure ulcers in the ICU.

Recently, growing evidence has shown that the BSS offers significant value beyond its original purpose. This scoring system could serve as a quick and non-invasive screening tool for identifying potential adverse outcomes across various populations. A significant association has been observed between BSS and overall mortality in patients with ischemic stroke, acute myocardial infarction, frailty, COVID-19, and those receiving care in cardiac intensive units (9–14). Ding et al. have shown that the BSS, with a cutoff point of 15 points, exhibits moderate validity for predicting stroke-associated pneumonia following spontaneous intracerebral hemorrhage (15). Also, considering pressure injurie is one of the significant risk factors for delirium, several studies have further explored the relationship between BSS and delirium in specific patient populations. Cheng et al. reported a significant negative correlation between admission BSS and delirium incidence among critically ill patients aged over 65 years old (16). In a cohort study involving 3,680 cases of ischemic stroke, after adjusting for confounding factors, a negative correlation was observed between the BSS and the risk of developing delirium (17). Currently, there is still a lack of studies specifically addressing the relationship between BSS and delirium risk among SICU patients. Therefore, this study aims to explore the association between BSS at ICU admission and delirium incidence in SICU patients, seeking a simple yet innovative method for early postoperative delirium intervention.

## 2 Methods

### 2.1 Data source

This study was a retrospective observational cohort study based on the Medical Information Mart for Intensive Care (MIMIC)-IV version 2.2 (v2.2). This database is an open-access resource containing information on approximately 299,712 adult patients, including 431,231 inpatients and 73,181 ICU admissions at Beth Israel Deaconess Medical Center (Boston, USA) from 2008 to 2019 (18). An institutional review board from Massachusetts Institute of Technology and Beth Israel Deaconess Medical Center approved the creation of the database (19). One of our authors obtained the necessary certification (MS: Certification Number: 66963718) and subsequently extracted relevant variables required for this research. As patient health information within this database has been anonymized, it was not necessary to obtain individual consent from each patient. This study followed the Strengthening the Reporting of Observational Studies in Epidemiology (STROBE) guidelines for reporting (20).

### 2.2 Study population

**Figure 1:**
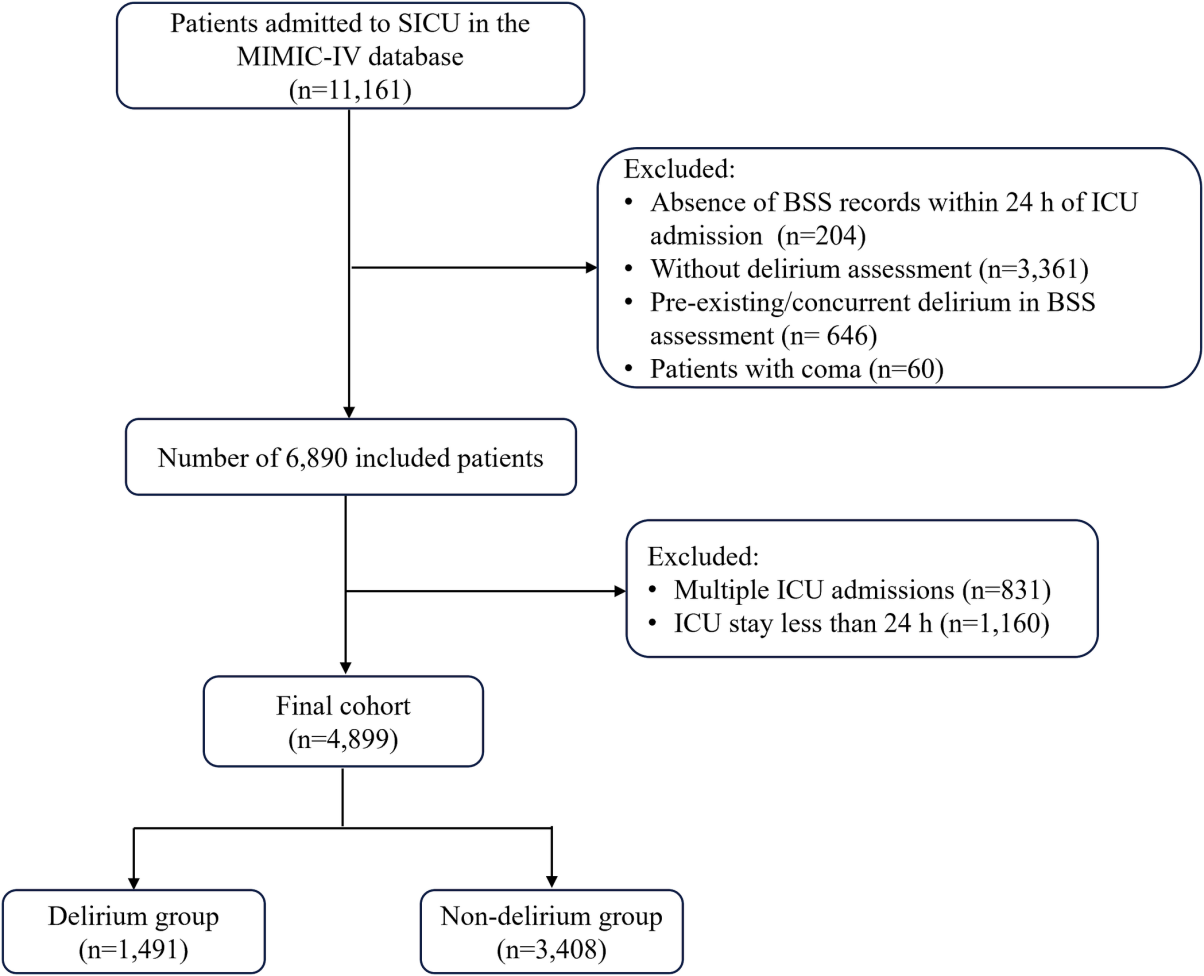
The flowchart of this study.

Patients were included if they met the following criteria: (1) admitted to the surgical intensive care unit; (2) patients with BSS documentation within 24 h of ICU admission; (3) patients with delirium assessment during ICU stay. The exclusion criteria were as follows: (1) patients with pre-existing or concurrent delirium in BSS assessment; (2) patients with coma; (3) multiple ICU admissions (only their first admission was analyzed); (4) ICU length of stay less than 24 h (Figure 1). The primary outcome of this study was the occurrence of delirium during ICU stay.

We defined the BSS records obtained within 24 h of ICU admission as the initial ICU admission BSS. Specificity, the BSS is a widely utilized tool for bedside nursing assessments in the ICU and it evaluates the risk of patients developing pressure ulcers based on six critical factors: sensory perception, activity level, mobility, moisture, nutritional status, and friction/shear (7, 8). Each factor is assigned a score ranging from 1 to 4, with the exception of the friction/shear component, which has a scoring range of 1 to 3. After individually scoring each category, the scores are summed to obtain a total score. A lower total score indicates an increased risk of pressure ulcer development during hospitalization (21). In the MIMIC-IV v2.2 database, delirium assessment was performed using the Confusion Assessment Method for ICU (CAM-ICU) score. According to the 2013 Society of Critical Care Medicine guidelines for pain, agitation, and delirium, CAM-ICU is the most effective tool for diagnosing and evaluating delirium in adult ICU patients (22). The assessment comprises four key features: (1) an acute onset of mental status changes or a fluctuating course; (2) inattention; (3) disorganized thinking; and (4) an altered level of consciousness. In this study, we define delirium as any episode of delirium that may occur during a patient’s ICU stay. This is clearly identified through a positive result (i.e., a CAM-ICU positive) obtained from at least one delirium assessment. A diagnosis of delirium (i.e., a CAM-ICU positive) is made if a patient exhibits with features (1) (2), along with either feature (3) or (4).

### 2.3 Variable extraction

We utilized structured query language (SQL) with PostgreSQL (version 15.2) to extract the following variables from the MIMV-IV v2.2 database: (1) demographic characteristics: age, race, and sex; (2) comorbidities: chronic pulmonary disease, congestive heart failure, acute myocardial infarction, dementia, cerebrovascular disease, liver disease, renal disease, diabetes, sepsis, and malignancy; (3) disease scores: Braden Skin Score, sequential organ failure assessment (SOFA), and Glasgow Coma Score (GCS); (4) the mean vital signs during first 24 h after ICU admissions: heart rate (HR), mean blood pressure, respiratory rate, temperature, and oxyhemoglobin saturation (SpO_2_); (5) the laboratory indicators during first 24 h: blood routine count, liver enzymes, renal indicators, electrolyte, and coagulation metrics. If a laboratory variable was measured multiple times, we selected the value indicating the greatest severity of illness. We also noted the ICU interventions during hospitalization including mechanical ventilation, renal replacement therapy, sedative medications, and vasopressor use.

### 2.4 Feature selection

Before investigating the correlation between BSS and delirium events, we first used machine learning algorithms for feature selection to highlight the importance of BSS in predicting delirium. We employed the Boruta algorithm, a well-known method that identifies key features by comparing their *Z* values with those of “shadow features” (23). In this approach, all real features are duplicated and randomized, followed by calculating *Z* values for each feature using a random forest model. Shadow feature *Z* values are generated by shuffling real features. If a real feature’s *Z* value is significantly higher than the maximum *Z* value of shadow features across multiple tests, it is deemed “important” (marked in green). Otherwise, it is classified as “unimportant” (marked in red). Through this feature selection, we elucidated the significant value of BSS in predicting delirium. Furthermore, it provided a more comprehensive reference basis for the inclusion of confounding factors in subsequent multivariable logistic regression analyses.

### 2.5 Statistical analyses

In this study, basic clinical characteristics of patients were compared between the delirium group and the non-delirium group. The differences in categorical variables were analyzed using the Fisher’s exact or chi-square tests and were presented as counts (percentages), while continuous variables were assessed through Student’s t-test or the Wilcoxon rank-sum test and were expressed as median (interquartile range).

**Figure 2:**
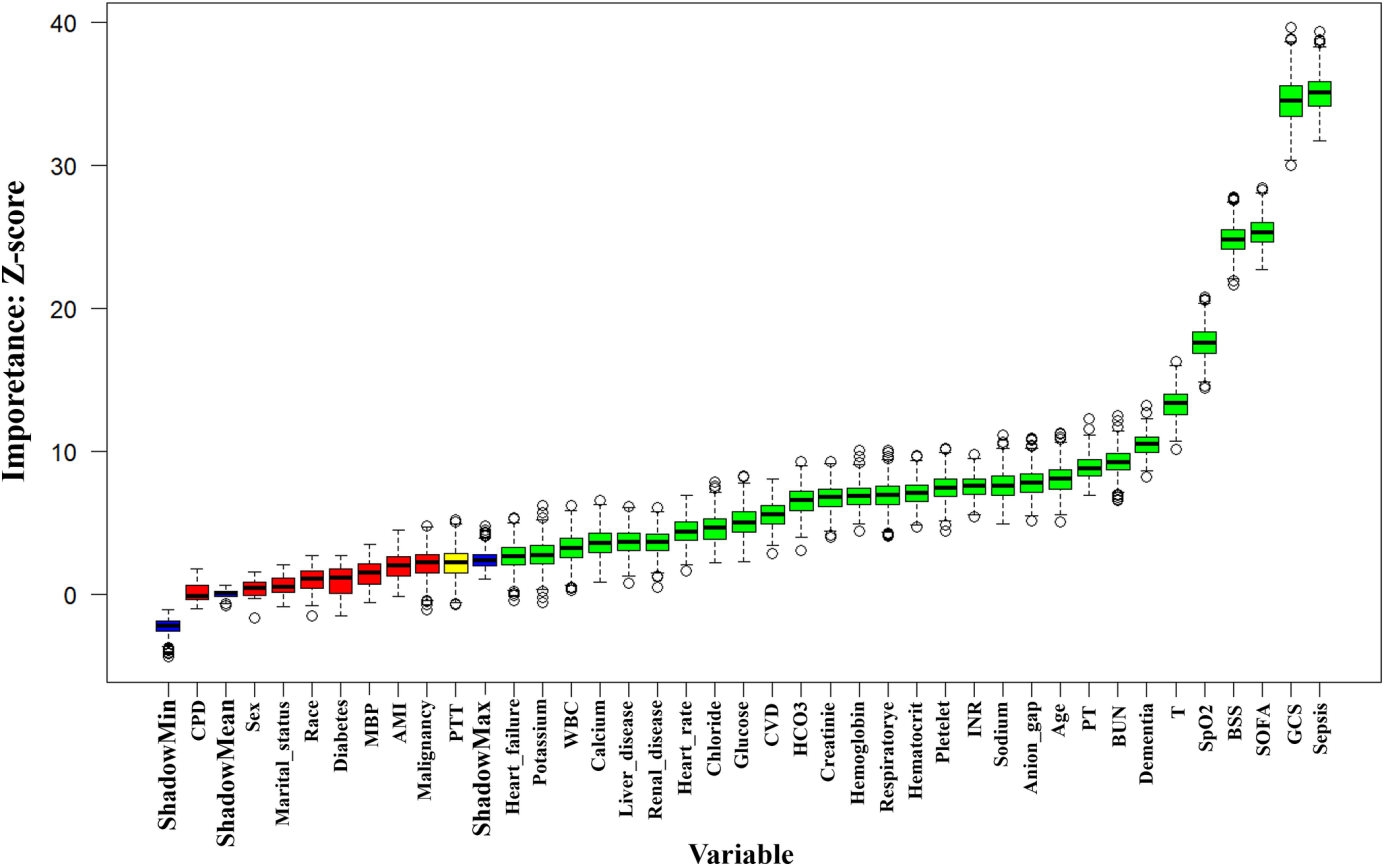
Feature selection utilizing the Boruta algorithm. The horizontal axis lists variable names, while the vertical axis shows their corresponding *Z* values. The box plot illustrates these *Z* values during model computation. Green boxes indicate important variables, and red boxes represent unimportant ones. BSS, Braden Scale Score; BUN, blood urea nitrogen; CPD, Chronic pulmonary disease; CVD, Cerebrovascular disease; GCS, Glasgow Coma Score; HCO_3_, Bicarbonate; ICU, intensive care unit; INR, international normalized ratio; MBP, mean blood pressure; MV, mechanical ventilation; PT, Prothrombin time; PTT, partial thromboplastin time; RRT, renal replacement therapy; SOFA, the Sequential Organ Failure Assessment; SpO_2_, oxyhemoglobin saturation; T, temperature; WBC, white blood cell.

To explore potential non-linear relationships between BSS and the risk of ICU delirium, the 3-knotted multivariable restricted cubic spline (RCS) regression analysis was conducted. We categorized BSS based on the cutoff point obtained from RCS models, using the lower BSS as the reference group. Then, we employed multivariable logistic regression to further assess the association between the continuous variable per one unit and the grouped BSS and the risk of delirium. Given the considerations of prior literature, clinical experience, and important variables through feature selection from the Boruta algorithms (as showed in Figure 2), we built three models in the RCS and logistic regression. Model 1 included only BSS, without any adjustments. Model 2 adjusted for sex, age, race, vital signs (HR, respiratory rate, temperature, and SpO_2_), and laboratory indicators (white blood cell, platelet, hemoglobin, glucose, creatinine, blood urea nitrogen, potassium, calcium, and international normalized ratio). Model 3, based on model I and model II, but further adjusted for congestive heart failure, cerebrovascular disease, dementia, renal disease, liver disease, sepsis, and GCS. Variance inflation factors (VIFs) were examined to evaluate multicollinearity among the variables ultimately included in the adjusted regression model, ensuring that all VIF values remained below 2.5 (Supplementary Table 1) (24).

Furthermore, stratified analyses were conducted to validate the consistency of BSS in predicting delirium among different subgroups, including sex, age (< 65 and ≥ 65 years), gender, ethnicity (White, Black, and others), congestive heart failure, cerebrovascular disease, liver disease, renal disease, sepsis, and dementia. The cumulative 90-day survival rates among grouped BSS patients (BSS < 16 and BSS ≥ 16) with or without delirium during hospitalization were compared by the Kaplan–Meier survival curves and evaluated using the Log-rank test. Additionally, given that delirium has been shown to be associated with an increased short-term mortality risk in SICU patients (3), we employed the mediation analysis to explore the potential mediating effects of delirium in the relationship between each BSS and the risk of 90-day mortality. Bootstrap resampling with 1,000 repetitions was utilized to assess the effect sizes. A significant effect was indicated when the 95% confidence interval did not include zero.

To ensure the robustness of the results, sensitivity analyses were performed in several scenarios. Firstly, patients who died during their ICU stay were excluded to minimize the influence of competing death outcomes on the risk of delirium. Secondly, considering that sepsis is a well-known major contributor to delirium in critically ill patients, we excluded individuals diagnosed with sepsis to mitigate the impact of confounding factors. Thirdly, as dementia is often linked to delirium in the ICU setting, we further excluded those diagnosed with dementia. Additionally, patients with a GCS score of 8 or lower upon ICU admission may affect the assessment of delirium or BSS; thus, this specific subset was excluded to enhance the reliability of our conclusions.

All analyses were conducted using R software (version 4.2.0) and Stata software (version 16). A two-sided *p*-value of less than 0.05 was deemed statistically significant.

## 3 Results

### 3.1 Baseline characteristics

According to the established inclusion and exclusion criteria, this study enrolled a total of 4,899 patients admitted to the SICU, among whom 1,491 (30.4%) were diagnosed with delirium during their ICU stay (Figure 1). The basic clinical characteristics of all participants are presented in Table 1. Compared to non-delirium patients, those with delirium were older and more likely to have comorbidities such as congestive heart failure, diabetes, cerebrovascular disease, dementia, renal disease, liver disease, and sepsis. Additionally, these patients exhibited more pronounced disturbances in vital signs upon admission and demonstrated significant abnormalities in initial laboratory indicators. Furthermore, patients with delirium had lower BSS and GCS while exhibiting higher SOFA scores, indicating more severe conditions. Additionally, a higher proportion of these patients required mechanical ventilation, vasopressor support, sedatives, and renal replacement therapy during ICU stay. In contrast, gender differences and the presence of chronic pulmonary disease, along with serum chloride and sodium levels, were not statistically significant (*p* > 0.05).

**Table 1:** Baseline characteristics of participants.

Variables	Overall (*n* = 4,899)	Non-delirium (*n* = 3,408)	Delirium (*n* = 1,491)	*p* value
Age (years)	64.89 (53.58, 75.97)	63.76 (52.57, 74.53)	67.47 (56.55, 78.60)	<0.001
Male (%)	2,594 (52.9%)	1,775 (52.1%)	819 (54.9%)	0.066
Marital status				0.047
Single	1,780 (36.3%)	1,208 (35.4%)	572 (38.4%)	
Married	2,222 (45.4%)	1,585 (46.5%)	637 (42.7%)	
Others	897 (18.3%)	615 (18.0%)	282 (18.9%)	
Ethnicity (%)				0.009
White	3,215 (65.6%)	2,278 (66.8%)	937 (62.8%)	
Black	499 (10.2%)	347 (10.2%)	152 (10.2%)	
Others	1,185 (24.2%)	783 (23.0%)	402 (27.0%)	
Comorbidities (%)				
Chronic pulmonary disease	1,130 (23.1%)	768 (22.5%)	362 (24.3%)	0.2
Congestive heart failure	866 (17.7%)	558 (16.4%)	308 (20.7%)	<0.001
Acute myocardial infarction	485 (9.9%)	292 (8.6%)	193 (12.9%)	<0.001
Diabetes	1,274 (26.0%)	832 (24.4%)	442 (29.6%)	<0.001
Cerebrovascular disease	1,583 (32.3%)	1,071 (31.4%)	512 (34.3%)	0.045
Liver disease	776 (15.8%)	471 (13.8%)	305 (20.5%)	<0.001
Renal disease	846 (17.3%)	543 (15.9%)	303 (20.3%)	<0.001
Malignancy	729 (14.9%)	534 (15.7%)	195 (13.1%)	0.019
Dementia	134 (2.7%)	61 (1.8%)	73 (4.9%)	<0.001
Sepsis	2,167 (44.2%)	1,148 (33.7%)	1,019 (68.3%)	<0.001
Vital signs				
Heart rate (min)	82.68 (72.32, 93.97)	81.72 (71.68, 93.12)	84.43 (74.38, 96.44)	<0.001
MBP (mmHg)	79.71 (72.90, 87.70)	80.00 (73.16, 88.13)	78.67 (72.25, 86.76)	<0.001
Respiratory rate (min)	18.18 (16.19, 20.71)	17.87 (16.04, 20.31)	18.94 (16.69, 21.52)	<0.001
Temperature (°C)	36.91 (36.72, 37.15)	36.88 (36.72, 37.10)	36.97 (36.74, 37.28)	<0.001
SPO_2_ (%)	97.13 (95.88, 98.45)	96.96 (95.76, 98.20)	97.65 (96.23, 98.94)	<0.001
Laboratory indicators				
WBC (10^9^/L)	10.80 (8.00, 14.50)	10.52 (7.80, 14.10)	11.25 (8.50, 15.30)	<0.001
Platelet (10^9^/L)	201.50 (148.50, 261.50)	204.00 (155.00, 265.75)	190.50 (131.00, 252.00)	<0.001
Hemoglobin (10^12^/L)	11.25 (9.50, 12.85)	11.35 (9.70, 12.90)	10.95 (9.15, 12.55)	<0.001
Hematocrit (%)	34.10 (29.30, 38.45)	34.35 (29.65, 38.60)	33.35 (28.10, 37.95)	<0.001
Anion gap (mmol/L)	15.00 (13.00, 17.00)	14.50 (12.50, 16.50)	15.00 (13.00, 17.50)	<0.001
Bicarbonate (mmol/L)	23.00 (21.00, 25.50)	23.50 (21.00, 25.50)	22.50 (20.00, 25.00)	<0.001
Sodium (mmol/L)	138.5 (136.0, 141.0)	138.5 (136.0, 140.5)	138.5 (136.0, 141.5)	0.064
Potassium (mmol/L)	4.05 (3.75, 4.40)	4.00 (3.75, 4.40)	4.10 (3.75, 4.50)	0.017
Creatinine (mg/dL)	0.90 (0.70, 1.30)	0.90 (0.70, 1.20)	1.00 (0.70, 1.50)	<0.001
BUN (mg/dL)	17.50 (12.00, 27.00)	16.50 (12.00, 25.00)	19.50 (13.50, 31.50)	<0.001
Calcium (mmol/L)	8.45 (8.00, 8.90)	8.45 (8.05, 8.90)	8.40 (7.95, 8.90)	0.005
Chloride (mmol/L)	104.0 (100.5, 107.0)	104.0 (101.0, 107.0)	104.0 (100.0, 107.0)	>0.9
Glucose (mmol/L)	133.0 (111.0, 163.0)	131.0 (110.0, 159.0)	139.50 (115.0, 173.5)	<0.001
Prothrombin time (s)	12.90 (11.70, 15.15)	12.90 (11.65, 14.35)	13.15 (11.95, 17.00)	<0.001
PTT (s)	30.30 (27.50, 35.10)	30.30 (27.50, 34.10)	30.30 (27.50, 37.95)	<0.001
INR	1.20 (1.10, 1.40)	1.20 (1.10, 1.30)	1.20 (1.10, 1.55)	<0.001
Scoring systems				
BSS	16.00 (14.00, 17.00)	16.00 (14.00, 18.00)	14.00 (13.00, 16.00)	<0.001
SOFA	3.00 (2.00, 6.00)	3.00 (1.00, 5.00)	5.00 (3.00, 8.00)	<0.001
GCS	15.00 (13.00, 15.00)	15.00 (14.00, 15.00)	14.00 (12.00, 15.00)	<0.001
ICU intervention				
Vasopressor use (%)	1,387 (28.3%)	685 (20.1%)	702 (47.1%)	<0.001
MV use (%)	1,894 (38.7%)	906 (26.6%)	988 (66.3%)	<0.001
RRT use (%)	325 (6.6%)	150 (4.4%)	175 (11.7%)	<0.001
Sedatives use (%)	2,151 (43.9%)	1,071 (31.4%)	1,080 (72.4%)	<0.001
ICU-stay (days)	2.51 (1.63, 4.75)	1.98 (1.39, 3.12)	5.17 (2.90, 9.97)	<0.001
Hospital-stay (days)	8.14 (4.65, 14.96)	6.73 (3.79, 11.35)	13.92 (8.01, 22.99)	<0.001
ICU mortality (%)	294 (6.0%)	171 (5.0%)	123 (8.2%)	<0.001
Hospital mortality (%)	561 (11.5%)	313 (9.2%)	248 (16.6%)	<0.001
30-day mortality (%)	669 (13.7%)	366 (10.7%)	303 (20.3%)	<0.001
90-day mortality (%)	917 (18.7%)	504 (14.8%)	413 (27.7%)	<0.001

Data are presented as a number with the percentage in parentheses, or as the median with the interquartile range in parentheses. BSS, Braden Scale Score; BUN, blood urea nitrogen; GCS, Glasgow Coma Score; ICU, intensive care unit; INR, international normalized ratio; MBP, mean blood pressure, MV, mechanical ventilation; PTT, partial thromboplastin time; RRT, renal replacement therapy; SOFA, the Sequential Organ Failure Assessment; SpO_2_, oxyhemoglobin saturation; WBC, white blood cell.

In terms of outcomes, patients with delirium experienced prolonged stays in the ICU and overall hospitalizations while demonstrating poorer prognoses—evidenced by elevated ICU mortality as well as in-hospital mortality and short-term mortality at 30 days and 90 days (all *p* < 0.001) (Table 1).

### 3.2 Feature selection

We employed machine learning algorithms for feature selection to determine the significance of these variables in predicting delirium. Figure 2 illustrated the results of feature selection based on the Boruta algorithm. In this algorithm, variables identified within the green area were recognized as important features and were recommended for retention in subsequent multivariable analyses, while those in the red area were considered irrelevant and could be discarded. The results revealed that the top four significant variables influencing delirium occurrence were sepsis, GCS, SOFA, and BSS. This further emphasized the potential value of BSS in forecasting delirium.

### 3.3 Associations between BSS and delirium

**Figure 3:**
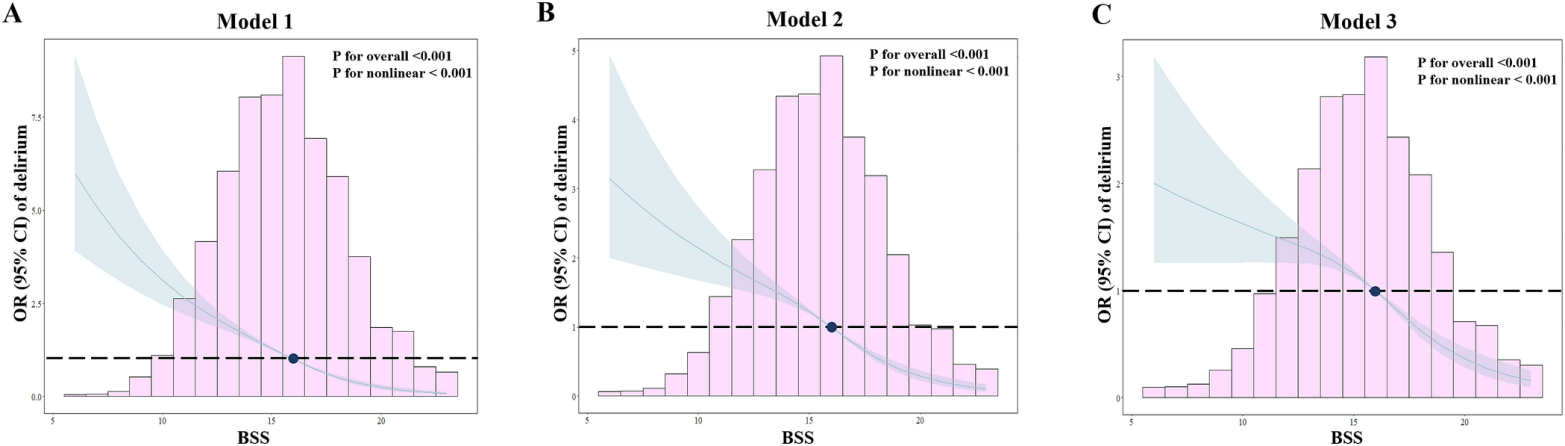
RCS analyses for the relationship between BSS and delirium. The horizontal dashed line indicates a hazard ratio of 1.0, with the cut-off value for BSS set at 16. **(A)** Model 1: no adjustments; **(B)** Model 2: sex, age, race, vital signs (HR, respiratory rate, temperature, and SpO_2_), and laboratory indicators (white blood cell, platelet, hemoglobin, glucose, creatinine, blood urea nitrogen, potassium, calcium, and international normalized ratio); **(C)**. Model 3: based on Model 1 and Model 2 and further adjusted for congestive heart failure, cerebrovascular disease, dementia, renal disease, liver disease, sepsis, and Glasgow Coma Score.

We explored the potential non-linear association between BSS and the occurrence of delirium in SICU patients through an analysis utilizing RCS models. As illustrated in Figure 3, we observed an L-shaped non-linear relationship between BSS and delirium across three different models. Notably, even after fully adjustment for confounding factors in Model 3, the non-linear relationship between BSS and delirium risk remained statistically significant (*p* for nonlinearity <0.001, *p* for overall <0.001). Through RCS analysis, we identified a threshold value of BSS at 16 for assessing delirium development: when BSS exceeded 16, the incidence of delirium significantly decreased. Consequently, we categorized the population into two groups based on their BSS values: those with BSS < 16 and those with BSS ≥ 16. We subsequently compared the relationships between continuous BSS and grouped BSS regarding delirium risk in multivariable logistic regression.

As illustrated in Table 2, our analysis of BSS as a continuous variable revealed that each unit increase was associated with a noteworthy reduction in the risk of delirium. Specifically, the odds ratios (OR) along with their 95% confidence intervals (CI) across three models were as follows: 0.78 (0.76–0.80), 0.82 (0.80–0.85), and 0.87 (0.84–0.89), respectively, all demonstrating statistical significance with *p* < 0.001. When analyzing as binary BSS, it was found that individuals with BSS ≥ 16 exhibited a significantly lower risk of delirium compared to those with BSS < 16 in Model 1 [OR (95% CI): 0.32 (0.29–0.37), *p* < 0.001]. Similar trends were also demonstrated in Model 2 [OR (95% CI): 0.42 (0.37–0.49), *p* < 0.001] and Model 3 [OR (95% CI): 0.53 (0.46–0.61), *p* < 0.001].

**Table 2:** The association between BSS and delirium.

BSS	Model 1 OR (95% CI)	*p* value	Model 2 OR (95% CI)	*p* value	Model 3 OR (95% CI)	*p* value
Continuous	0.78 (0.76–0.80)	<0.001	0.82 (0.80–0.85)	<0.001	0.87 (0.84–0.89)	<0.001
Category						
Q1 (BSS < 16)	Ref.		Ref.		Ref.	
Q2 (BSS ≥ 16)	0.32 (0.29–0.37)	<0.001	0.42 (0.37–0.49)	<0.001	0.53 (0.46–0.61)	<0.001

Model 1: no adjustments; Model 2: sex, age, race, vital signs (HR, respiratory rate, temperature, and SpO_2_), and laboratory indicators (white blood cell, platelet, hemoglobin, glucose, creatinine, blood urea nitrogen, potassium, calcium, and international normalized ratio); Model 3: based on Model 1 and Model 2 and further adjusted for congestive heart failure, cerebrovascular disease, dementia, renal disease, liver disease, sepsis, and Glasgow Coma Score. BSS, Braden Scale Score; OR, odds ratio; CI, confidence interval; Ref., reference.

### 3.4 Subgroup analysis

**Figure 4:**
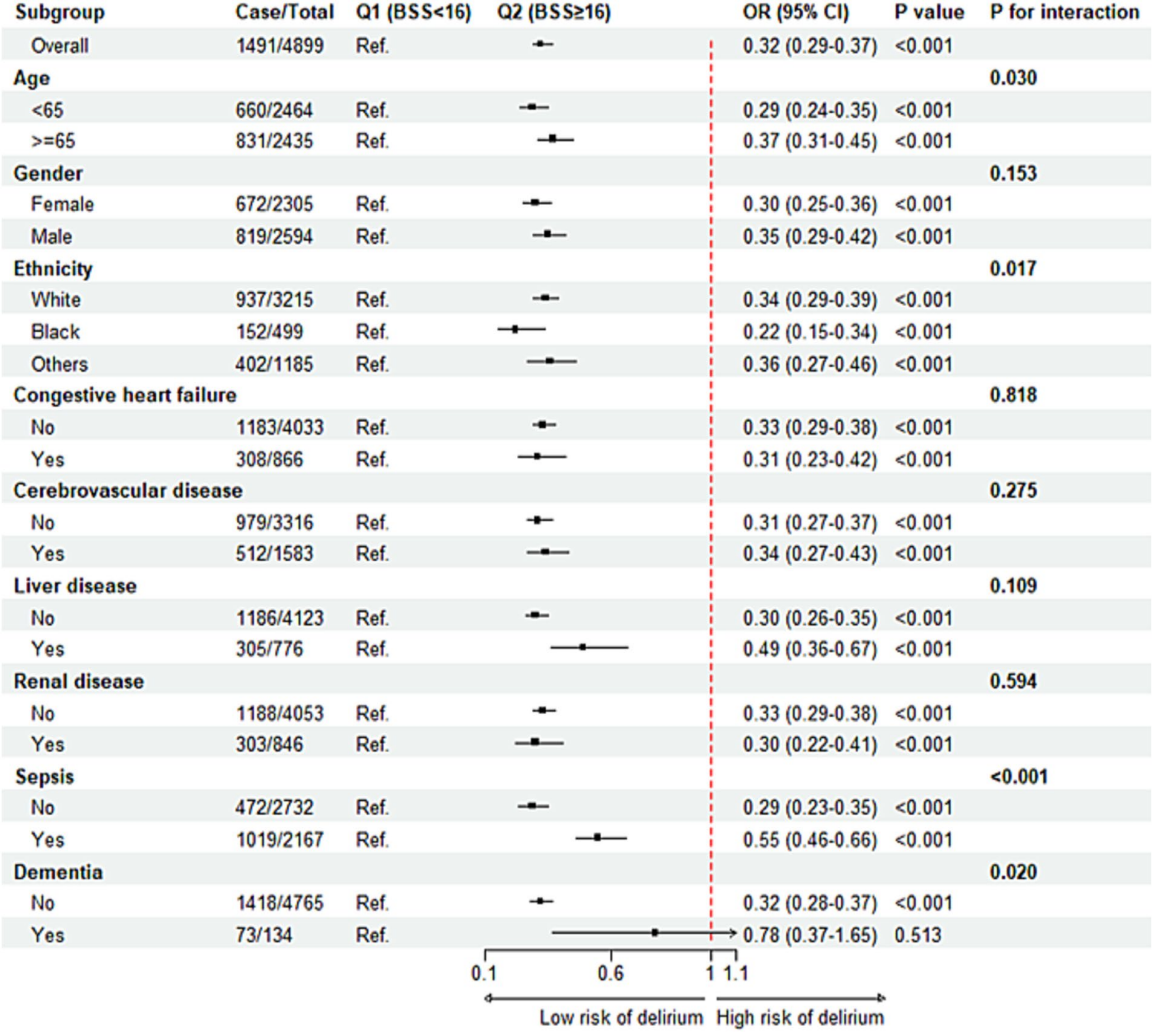
Subgroup analysis regarding the association between BSS and the risk of delirium. BSS, Braden Scale Score; OR, odds ratio, CI, confidence interval.

The subgroup analysis was conducted to explore the correlation between BSS and ICU delirium in specific populations (Figure 4). In subgroups stratified by age, sex, race, congestive heart failure, cerebrovascular diseases, liver disease, kidney disease, and sepsis, we observed a significantly reduced risk of association between BSS and delirium in individuals with a BSS score greater than or equal to 16. However, within the subgroup of patients diagnosed with dementia, although there was a positive correlation between BSS and delirium, this relationship did not achieve statistical significance [OR (95% CI): 0.78 (0.37–1.65), *p* = 0.513]. Furthermore, the interaction analysis revealed a significant interaction among age, race, sepsis, and dementia subgroups. This indicated that the assessment value of BSS for delirium varies across these different subgroup populations.

### 3.5 Sensitivity analysis

The sensitivity analyses conducted in this study confirmed the robustness of the primary findings. Firstly, the exclusion of 294 patients who died in the ICU did not significantly diminish the statistical association between BSS and delirium (Supplementary Table 2). Furthermore, after excluding 2,167 cases of sepsis, the multivariable logistic regression analysis still indicated a significant correlation between elevated BSS and a reduced risk of delirium in SICU patients (Supplementary Table 3). Moreover, even after excluding 134 patients with dementia, the results from the sensitivity analysis revealed that lower BSS remained an independent risk factor for delirium among SICU patients (Supplementary Table 4). Lastly, after excluding 342 patients with GCS scores ≤8 upon admission, results still showed that higher BSS was associated with reduced instances of delirium (Supplementary Table 5).

### 3.6 The interaction among BSS, delirium and short-term mortality

**Figure 5:**
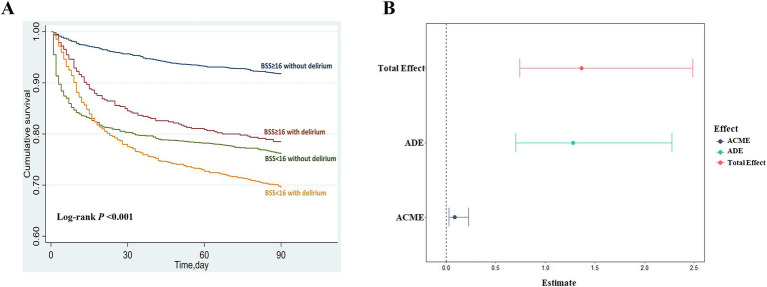
Relationship between BSS, delirium, and short-term mortality. **(A)** Patients were categorized into four groups based on their admission BSS (BSS < 16 or BSS ≥ 16) and the presence of delirium. The Kaplan–Meier curves showed the cumulative 90-day survival rates for each group, with differences analyzed using the log-rank test. **(B)** The total effect, including direct and indirect effects, was assessed using mediation analysis with 1,000 bootstrap iterations. A significant effect was indicated if the 95% confidence interval (CI) did not include zero. ADE average direct effect, ACME average causal mediation effect.

Overall, patients with a BSS score of 16 or higher demonstrated significantly lower short-term mortality compared to those with a BSS score of less than 16 (Supplementary Figure 1). Additionally, considering that patients with delirium may also have higher short-term mortality (Table 1), we investigated how different grouped BSS and the presence of delirium affect this risk, as well as the indirect effect of delirium on the relationship between BSS and patient prognosis. The Kaplan–Meier analysis revealed that the 90-day survival rate for patients with lower BSS and delirium was significantly lower than that of those without these conditions (Figure 5A). The cumulative survival rates across the four groups were as follows: BSS <16 with delirium, BSS <16 without delirium, BSS ≥16 with delirium, and BSS ≥16 without delirium: 69.5% vs. 76.1% vs. 78.5% vs. 91.7%, respectively (Log-rank *p* < 0.001). The mediation analysis indicated that delirium served as a mediator in the relationship between BSS and 90-day mortality [Total effect: 1.366 (0.740–2.490), *p* < 0.001; indirect effect: 0.088 (0.029–0.220), *p* < 0.001; direct effect: 1.278 (0.703–2.270), *p* < 0.001], accounting for approximately 6.4% of the total effect (Figure 5B).

## 4 Discussion

To our knowledge, this is the first study focusing on the association between admission BSS and the risk of delirium among patients in surgical ICU settings. We performed a retrospective analysis using a large public medical database to explore this relationship. The preliminary feature selection highlighted the significance of BSS in predicting delirium occurrence. Further RCS analysis indicated a non-linear relationship between BSS and delirium. By analyzing the cutoff points of RCS curves, patients with delirium were divided into two groups based on their admission BSS. After adjusting for various covariables, multivariable logistic regression results showed that both linear and grouped BSS suggested a close association between lower initial BSS and increased risk of delirium. Subgroup and sensitivity analyses further confirmed the robustness of these conclusions under different contexts. In addition, lower BSS were associated with an increased all-cause 90-day mortality in patients, with delirium contributing approximately 6.4% to this total effect. Our research indicated a positive correlation between BSS upon admission and the risk of delirium in critically ill surgical patients. Specifically, patients with an initial BSS score below 16 exhibited a significantly increased risk of developing delirium, as well as a higher incidence of short-term poor prognosis.

Critically ill patients in the SICU are often at an elevated risk of developing delirium (4). In addition to the common risk factors for delirium observed in the general ICU, SICU patients encounter unique perioperative factors that further heighten their susceptibility to this condition. These distinctive factors include major cardiac and pulmonary surgeries, operative time, anesthesia medications, and pain stimuli experienced during perioperative (3, 4). Previous researches have confirmed that delirium is an independent predictor of excess death, length of stay, cost of care, and acquired dementia (4). Our study also demonstrated that patients experiencing delirium presented with more severe conditions, longer hospital stays, and an elevated risk of mortality in the ICU, during hospitalization, and at 90 days post-discharge. Early identification and management of delirium are crucial for enabling prompt interventions that may reverse or alleviate its harmful effects. Despite various predictive models for delirium, these scales often involve numerous parameters and additional laboratory tests, complicating clinical application (5). Furthermore, their specificity may be insufficient for patients in the SICU. Consequently, reliable and convenient predictive indicators are still needed for the early identification of high-risk populations susceptible to delirium. The BSS is a widely used tool in nursing assessment for evaluating the risk of pressure ulcer in patients (6). Most critically ill patients undergo a BSS evaluation soon after ICU admission, making it accessible and easy to use in clinical practice. Recently, a multicenter cohort study focusing on older adults identified the BSS as an independent predictor of ICU delirium (16). This finding indicated that the BSS not only reflects skin conditions but also possesses predictive value for delirium. Therefore, it is recommended to incorporate the BSS into management strategies for delirium in elderly patients (16). Similarly, a retrospective analysis of 3,680 adult ICU patients with ischemic stroke revealed that lower Braden scores were strongly correlated with a significantly increased risk of delirium (17). This study established a cut-off score of 16 for the Braden Scale to identify individuals at high risk for delirium. Herein, consistent with previous studies, we confirmed a positive connection between lower BSS and the higher risk of developing ICU delirium, establishing a cutoff value of 16 for stratified patients. The findings underscored the necessity for increased vigilance regarding delirium risk in patients with low BSS upon admission and supported earlier intervention and management.

Some studies have revealed the correlation between BSS upon admission and patient prognosis (9–14). A retrospective cohort study of adult patients from the Mayo Clinic suggested that the BSS documented on admission was inversely associated with in-hospital mortality in patients in the cardiac intensive care units (CICU) (12). Moreover, the six individual BSS sub-scores were also inversely associated with hospital mortality after full adjustment. This finding suggested that BSS may serve as a rapid, noninvasive screening tool for identifying poor outcomes in CICU patients, potentially due to its ability to detect frail individuals (13). Also, Nygaard et al. found that in older acutely admitted medical patients, both the BSS and Clinical Frailty Scale were linked to 90-day mortality, with frail patients’ risk of death partially influenced by their BSS status (11). Another study has shown that the Braden Scale functioned as an independent predictor of 30-day mortality in critically ill patients suffering from ischemic stroke, demonstrating robust predictive performance with an area under the receiver operating characteristic curve of 0.71 (9). Similar to these researches, our study revealed that patients with a BSS score below 16 had a significantly higher mortality rate within 30 days and 90 days compared to those with a BSS score of 16 or above (Supplementary Figure 1). When categorizing patients into four groups based on their BSS and the presence of delirium, survival curves revealed that patients in lower BSS group who also exhibited delirium experienced the lowest survival rates (68.1%). In contrast, patients who presented with higher admission BSS and did not exhibit delirium demonstrated the most favorable prognosis, achieving a survival rate of 90.8% at 90 days. Furthermore, considering the significant influence of delirium on short-term mortality in patients, we performed a mediation analysis to elucidate the interaction with prognosis more clearly. Our findings suggested that BSS played a predominant directly impact on determining 90-day mortality among SICU patients, while the indirect effect mediated by delirium contributed only approximately 6%. Interestingly, Li et al. investigated the interactions among BSS levels, acute kidney injury (AKI), and long-term all-cause mortality in patients with acute coronary syndrome (25). They found that the indirect effect of the BSS on long-term all-cause mortality, mediated by AKI, accounted for 30%. This indicated that the impact of BSS on mortality among different patient populations may be mediated by specific indirect factors, while the duration of follow-up may also contribute to variations in this effect. In addition, our subgroup analysis showed no statistically significant association between BSS and delirium in dementia patients. This finding can be explained by several factors. First, the sample size of only 134 dementia patients may have weakened statistical power, rendering the differences insignificant. Second, the cognitive overlap between dementia and delirium makes it challenging to identify episodes of delirium in dementia patients, which may also affect the results. Therefore, future research should further explore the application value of BSS in assessing delirium within the dementia population.

Some potential mechanisms may elucidate the significant association between BSS upon admission and delirium in SICU patients. Firstly, the BSS is regarded as an effective indicator for assessing the risk of pressure ulcers in patients (8). A lower BSS indicated an increased risk of developing a pressure injury. Recent studies have investigated the relationship between delirium and pressure injuries, confirming that pressure injury is a significant risk factor for delirium (26, 27). Therefore, it is plausible that BSS may indirectly contribute to the occurrence of delirium by increasing the risk of pressure ulcers. Secondly, the Braden Skin scoring metric reflected, to a certain extent, the frailty status of patients, encompassing aspects such as their nutritional condition and mobility function. Previous studies have indicated that BSS could be considered as an important tool for identifying frailty (11, 13). Consequently, a lower BSS signified a more pronounced state of frailty in patients, thereby increasing the risk of delirium. In addition, sub-scores derived from the Braden Skin provided a comprehensive assessment of patients in various dimensions, including perception, mobility, activity, and nutritional status. Typically, lower BSS correlated with more severe conditions that may necessitate intensified therapeutic interventions in the ICU setting. These interventions may include mechanical ventilation, sedatives, prolonged bed rest, and associated complications like sepsis. Such treatment modalities and their comorbidities significantly heighten the likelihood of delirium during ICU stays (4). Furthermore, if a patient’s ability to perceive pain and stress is impaired, it may suggest cognitive dysfunction, potentially leading to lower BSS. This condition might further increase vulnerability to psychiatric disturbances such as delirium.

This study has several limitations. Firstly, it was an observational retrospective analysis that primarily explored the correlation between BSS and delirium without establishing a causal relationship. To enhance the robustness of our findings, we excluded patients with missing delirium assessment records, which may introduce selection bias. Future prospective studies should prioritize comprehensive data collection and dynamic monitoring of delirium events to reduce exclusions due to missing information. Secondly, despite significant efforts to gather clinical variables, potential bias from unaccounted confounders remains. Sedative use and the type of surgeries prior to delirium onset are recognized risk factors for delirium (28); however, we could not perform further adjusted analyses due to a lack of detailed data from the database. Also, the limitations of retrospective study hindered our in-depth analysis of delirium subtypes, potentially affecting our comprehensive understanding of this condition. Notably, while the BSS is a widely used subjective nursing assessment tool with acceptable interrater reliability, attention must be paid to potential interobserver variability. Implementing standardized training protocols and structured assessment procedures could improve measurement consistency and enhance clinical evaluation validity. Finally, this study focused on SICU patients; thus, its findings may not apply to patients in non-surgical ICUs or those in general wards. Future research should include diverse ICU settings and non-critical patients for broader applicability of results.

## 5 Conclusion

In summary, a lower admission BSS was found to be independently and positively correlated with an increased risk of delirium in SICU patients. Individuals with a BSS below 16 exhibited a heightened risk for both delirium and short-term mortality, necessitating increased vigilance and timely intervention from clinical staff. Our findings suggest that the BSS not only serves as a risk indicator for pressure injuries but also functions as an effective tool for classifying and managing delirium. Nevertheless, further prospective studies are necessary to validate these findings.

## Data Availability

The data are available on the MIMIC-IV website at https://mimic.physionet.org/, further inquiries can be directed to the corresponding author.
