# Supplementary material for: Association between admission Braden Skin Score and delirium in surgical intensive care patients: an analysis of the MIMIC-IV database
Source: Front Neurol. 2025 Apr 14;16:1555166. doi: 10.3389/fneur.2025.1555166 (PMC12036481; doi:10.3389/fneur.2025.1555166)
Supplement: Supplementary file 1 [file Data_Sheet_1.docx]

**Association Between Admission Braden Skin Score and Delirium in Surgical Intensive Care Patients: An Analysis of the MIMIC-IV Database**

**Supplementary Materials**


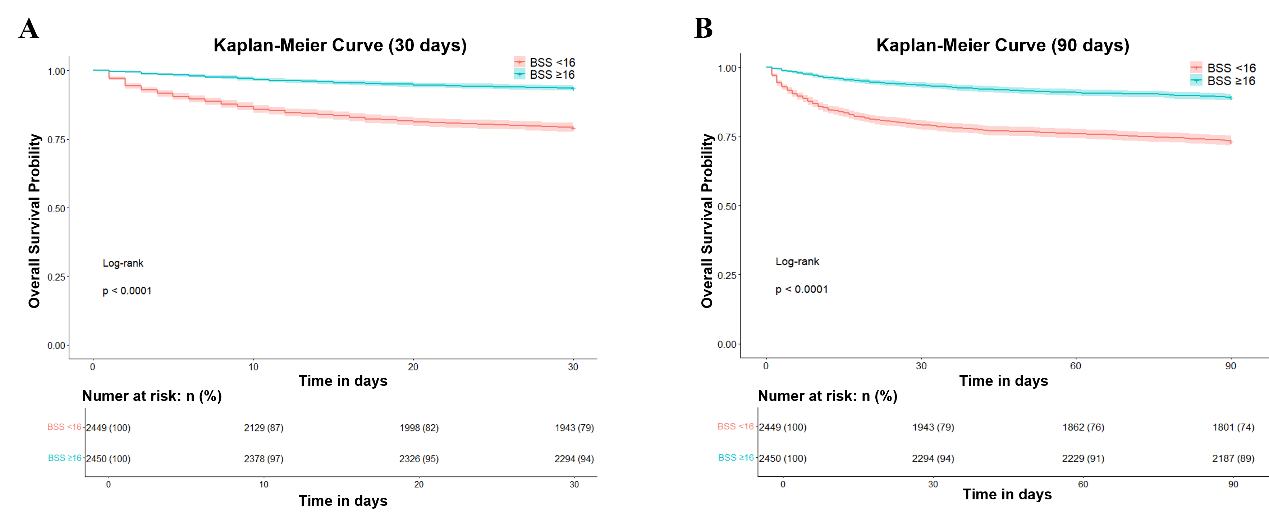


**Supplementary Figure 1**. Kaplan–Meier curves of short-term cumulative survival rates across BSS groups. **A.** 30-day survival. **B.** 90-day survival.

**Supplementary Table 1.** The variance inflation factor for all covariables of fully adjusted model.

| **Variable** | **VIF** |
| --- | --- |
| Braden Scale Score | 1.29 |
| Sex | 1.10 |
| Race | 1.05 |
| Age | 1.34 |
| Cerebrovascular disease | 1.22 |
| Dementia | 1.04 |
| Renal disease | 1.65 |
| Liver disease | 1.37 |
| Sepsis | 1.25 |
| Congestive heart failure | 1.24 |
| Glasgow Coma Score | 1.12 |
| White blood cell (10^9^/L) | 1.10 |
| Calcium (mmol/L) | 1.14 |
| Potassium (mmol/L) | 1.25 |
| Glucose (mg/dL) | 1.08 |
| Creatinine (mg/dL) | 2.10 |
| Hemoglobin (10^12^/L) | 1.44 |
| Blood urea nitrogen (mg/dL) | 2.10 |
| International normalized ratio | 1.17 |
| Platelet (10^9^/L) | 1.22 |
| Heart rate (min) | 1.38 |
| Respiratory (min) | 1.31 |
| Temperature (°C) | 1.15 |
| SpO_2_ (%) | 1.22 |

SpO_2_, oxyhemoglobin saturation.

**Supplementary Table 2.** The association between BSS and delirium after excluding patients who died during their ICU stay (n=4,605).

| **BSS** | **Model 1**  **OR (95%CI)** | **P value** | **Model 2**  **OR (95%CI)** | **P value** | **Model 3**  **OR (95%CI)** | **P value** |
| --- | --- | --- | --- | --- | --- | --- |
| Continuous | 0.76 (0.74-0.78) | <0.001 | 0.80 (0.78-0.83) | <0.001 | 0.85 (0.82-0.87) | <0.001 |
| Category |  |  |  |  |  |  |
| Q1 (BSS<16) | Ref. |  | Ref. |  | Ref. |  |
| Q2 (BSS≥16) | 0.31 (0.27-0.36) | <0.001 | 0.41 (0.36-0.47) | <0.001 | 0.51 (0.44-0.59) | <0.001 |

Model 1: no adjustments; Model 2: adjusted for sex, age, race, vital signs (HR, respiratory rate, temperature, and SpO_2_), and laboratory indicators (white blood cell, platelet, hemoglobin, glucose, creatinine, blood urea nitrogen, potassium, calcium, and international normalized ratio); **C.** Model 3: based on Model 1 and Model 2 and further adjusted for congestive heart failure, cerebrovascular disease, dementia, renal disease, liver disease, sepsis, and Glasgow Coma Score. BSS Braden Scale Score, OR odds ratio, CI confidence interval, Ref. reference.

**Supplementary Table 3.** The association between BSS and delirium after excluding patients with sepsis (n=2,732).

| **BSS** | **Model 1**  **OR (95%CI)** | **P value** | **Model 2**  **OR (95%CI)** | **P value** | **Model 3**  **OR (95%CI)** | **P value** |
| --- | --- | --- | --- | --- | --- | --- |
| Continuous | 0.76 (0.73-0.79) | <0.001 | 0.79 (0.76-0.83) | <0.001 | 0.82 (0.78-0.86) | <0.001 |
| Category |  |  |  |  |  |  |
| Q1 (BSS<16) | Ref. |  | Ref. |  | Ref. |  |
| Q2 (BSS≥16) | 0.29 (0.23-0.35) | <0.001 | 0.36 (0.29-0.45) | <0.001 | 0.42 (0.34-0.53) | <0.001 |

Model 1: no adjustments; Model 2: adjusted for sex, age, race, vital signs (HR, respiratory rate, temperature, and SpO_2_), and laboratory indicators (white blood cell, platelet, hemoglobin, glucose, creatinine, blood urea nitrogen, potassium, calcium, and international normalized ratio); **C.** Model 3: based on Model 1 and Model 2 and further adjusted for congestive heart failure, cerebrovascular disease, dementia, renal disease, liver disease, and Glasgow Coma Score. BSS Braden Scale Score, OR odds ratio, CI confidence interval, Ref. reference.

**Supplementary Table 4.** The association between BSS and delirium after excluding patients with dementia (n=4,765).

| **BSS** | **Model 1**  **OR (95%CI)** | | **P value** | **Model 2**  **OR (95%CI)** | **P value** | **Model 3**  **OR (95%CI)** | **P value** |
| --- | --- | --- | --- | --- | --- | --- | --- |
| Continuous | 0.78 (0.76-0.80) | | <0.001 | 0.82 (0.80-0.85) | <0.001 | 0.87(0.84-0.89) | <0.001 |
| Category | |  |  |  |  |  |  |
| Q1(BSS<16) | Ref. | |  | Ref. |  | Ref. |  |
| Q2(BSS≥16) | 0.32 (0.28-0.37) | | <0.001 | 0.42 (0.37-0.48) | <0.001 | 0.52  (0.45-0.60) | <0.001 |

Model 1: no adjustments; Model 2: adjusted for sex, age, race, vital signs (HR, respiratory rate, temperature, and SpO_2_), and laboratory indicators (white blood cell, platelet, hemoglobin, glucose, creatinine, blood urea nitrogen, potassium, calcium, and international normalized ratio); **C.** Model 3: based on Model 1 and Model 2 and further adjusted for congestive heart failure, cerebrovascular disease, sepsis, renal disease, liver disease, sepsis, and Glasgow Coma Score. BSS Braden Scale Score, OR odds ratio, CI confidence interval, Ref. reference.

**Supplementary Table 5.** The association between BSS and delirium after excluding patients with GCS≤8 (n=4,557).

| **BSS** | **Model 1**  **OR (95%CI)** | | **P value** | **Model 2**  **OR (95%CI)** | **P value** | **Model 3**  **OR (95%CI)** | **P value** |
| --- | --- | --- | --- | --- | --- | --- | --- |
| Continuous | 0.77 (0.75-0.79) | | <0.001 | 0.81  (0.79-0.83) | <0.001 | 0.84  (0.82-0.87) | <0.001 |
| Category | |  |  |  |  |  |  |
| Q1(BSS<16) | Ref. | |  | Ref. |  | Ref. |  |
| Q2(BSS≥16) | 0.33 (0.28-0.37) | | <0.001 | 0.42 (0.36-0.49) | <0.001 | 0.50  (0.43-0.58) | <0.001 |

Model 1: no adjustments; Model 2: adjusted for sex, age, race, vital signs (HR, respiratory rate, temperature, and SpO_2_), and laboratory indicators (white blood cell, platelet, hemoglobin, glucose, creatinine, blood urea nitrogen, potassium, calcium, and international normalized ratio); **C.** Model 3: based on Model 1 and Model 2 and further adjusted for congestive heart failure, cerebrovascular disease, dementia, renal disease, liver disease, sepsis, and Glasgow Coma Score. BSS Braden Scale Score, OR odds ratio, CI confidence interval, Ref. reference.
